# Preferences for seeking effort or reward information bias the willingness to work

**DOI:** 10.1038/s41598-022-21917-7

**Published:** 2022-11-14

**Authors:** Tanja Müller, Masud Husain, Matthew A. J. Apps

**Affiliations:** 1 Department of Experimental Psychology, University of Oxford, Oxford, UK; 2 Wellcome Centre for Integrative Neuroimaging, University of Oxford, Oxford, UK; 3 Nuffield Department of Clinical Neurosciences, University of Oxford, Oxford, UK; 4 Centre for Human Brain Health, School of Psychology, University of Birmingham, Birmingham, UK; 5 Institute for Mental Health, School of Psychology, University of Birmingham, Birmingham, UK; 6 Present Address: Zurich Center for Neuroeconomics, Department of Economics, University of Zurich, Zurich, Switzerland

**Keywords:** Psychology, Fatigue, Cognitive neuroscience, Motivation

## Abstract

Research suggests that the temporal order in which people receive information about costs and benefits whilst making decisions can influence their choices. But, do people have a preference for seeking information about costs or benefits when making effort-based decisions, and does this impact motivation? Here, participants made choices about whether to exert different levels of physical effort to obtain different magnitudes of reward, or rest for low reward. Prior to each effort-based choice, they also had to decide which information they wanted to see first: how much physical effort would be required, or how large the reward would be. We found no overall preference for seeking reward or effort information first, but motivation did change when people saw reward or effort information first. Seeking effort information first, both someone’s average tendency to do so and their choice to see effort first on a given trial, was associated with reductions in the willingness to exert higher effort. Moreover, the tendency to prefer effort information first was associated with reduced vigorous exercise and higher levels of fatigue in everyday life. These findings highlight that preferences for seeking effort information may be a bias that reduces people’s willingness to exert effort in the lab and in everyday life.

## Introduction

Motivation is often characterised as a cost–benefit trade-off, where people decide whether an amount of effort is ‘worth it’ for an offered amount of reward^1–5^. Decades of research have shown that people conform to a ‘law of less effort’ when making such effort-based decisions, avoiding higher effort unless it is associated with much larger rewards^2,4–7^. Effort-based decision-making paradigms have provided important insights into variability in motivation in the healthy population, and into pathological reductions in motivation in a range of clinical disorders^1,4,8–17^. However, this work has ignored a potentially important step in theoretical accounts of motivation, that may have a significant impact on how willing people are to exert effort in everyday life^10^. Before choosing whether to act we must first gather information about the rewards that can be obtained and the effort required to obtain them^18–20^. How people seek this information might significantly bias people’s willingness to exert effort for reward^18–20^.

Research has shown that people’s preferences for seeking different types of information can have powerful influences on their behaviour and welfare^21,22^. A number of biases pervade how we seek or avoid information (e.g. the information seeking bias, the optimism bias or confirmation bias). One prominent example is the information seeking bias—seeking information even when it may have little immediate utility for actions—with a powerful influence on behaviour. Variability in this bias and others that affect information seeking (for reviews see^21,22^) has been proposed to be a marker, symptom or even partial cause of psychiatric disorders such as depression and anxiety^21,22^. Thus, the extent to which people seek different types of information may be a crucial factor underlying typical and impaired behaviour. However, voluntary information seeking about effort has rarely been examined^23^, and thus whether motivation changes depending on people’s preferences for knowing how effortful or rewarding an act will be is unknown.

Strikingly, the willingness to exert effort appears to be a stable characteristic that is linked to individual differences between people^10,13,14,17^. Previous research has shown that a reduced willingness to exert effort is present in a range of neurological and psychiatric disorders, compared to the typical population^1^. Moreover, in the healthy population the willingness to exert effort is associated with psychiatric traits^14,24^. Notably, fatigue—a feeling of exhaustion that reduces levels of daily activity such as exercise^5,25–27^—is strongly associated with a reduced willingness to exert effort both in the healthy population and in a range of clinical conditions^17,25^. However, it is unclear whether this association between fatigue and motivation may also be linked to distinct patterns of seeking information that guide effort-based decisions.

Although people’s tendency to be averse to effort is relatively stable, the willingness to exert a given amount of effort for a particular reward can also change from moment-to-moment. As levels of fatigue fluctuate, or as there are changes in the reward environment, people shift whether they are willing to exert effort^2,5,28,29^. However, many of the factors underlying this variability have yet to be explored. Notably, other forms of decisions appear to be malleable based on how people seek or are presented with information that guides decisions^30–35^. Classical accounts in psychology suggest that persuasion to undertake actions depends on what kind of information that guides choices people are told first^36^. The order in which people are presented with information, such as items, monetary rewards or risks, or the order they voluntarily attend to information with eye movements, can bias other non-effort based economic decisions. This includes decisions under uncertainty, on whether to wait longer for a higher monetary reward rather than to choose a lower, more immediate reward, or choices about which item to choose among alternatives^19,37–40^. However, whether moment-to-moment variability in the seeking of information leads to similar variability in the willingness to exert effort is unknown.

Theories of motivation—such as those based on opportunity costs or motivational intensity—highlight that people devalue rewards by effort and that motivation depends on the costs and benefits of different courses of action^41,42^. However, recent evidence has demonstrated that if people are explicitly instructed about effort cost or reward information first, it can change their willingness to exert effort^43^. Although existing theories had posited that higher levels of fatigue might influence attention towards different bodily signals or how willing people are to switch to other activites^5,30^, the notion that simply seeing one of these sources of information first would change motivation had not been predicted. In these experiments^43^, people made a series of choices on whether to perform arithmetic calculations that varied in effort (easy versus hard) for varying amounts of rewards (small versus large). Crucially, information about the difficulty of the task and the magnitude of the reward (points) on offer was presented *consecutively,* with either the difficulty cue or reward cue presented first. When reward information was presented first, people were more likely to accept the offer, compared to when effort was presented first on a given trial. This has been suggested to be linked to neurocognitive mechanisms in the prefrontal cortex, where information presented first becomes prioritised and weighted more heavily into subsequent behaviour. Thus, even though people process and have access to exactly the same information, knowing the effort cost first reduces motivation. However, it is unclear if this impact of the temporal order of effort and reward information extends to situations where people can freely choose which information they receive first and to situations in which decisions on the expenditure of physical effort have to be made. Here, we predicted that when people seek effort information first, they will be less willing to exert effort.

**Figure 1:**
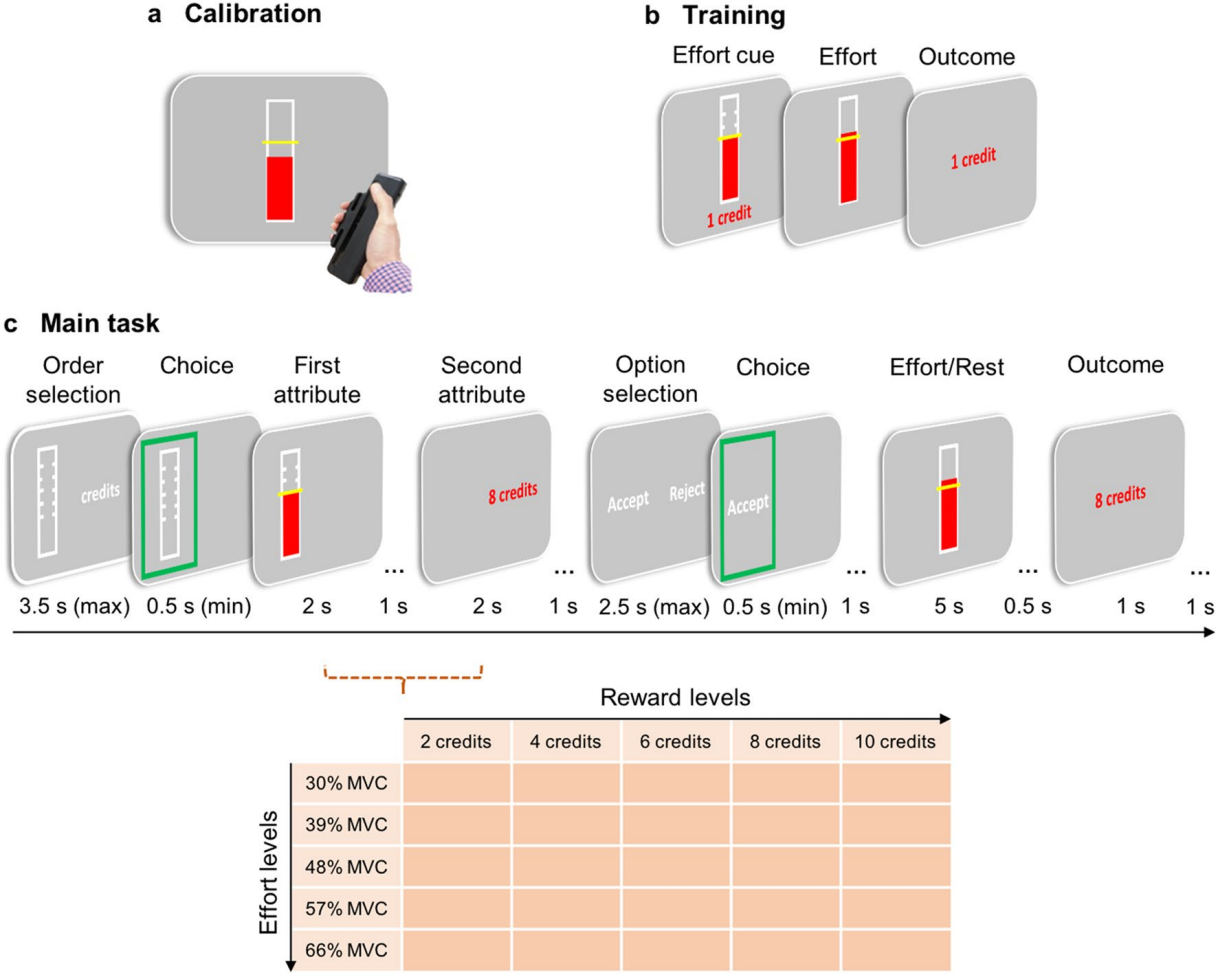
**Trial structure and experimental design.** (**a**) Each participant’s maximum voluntary contraction (MVC) was obtained by asking them to exert as much force as possible on a handheld dynamometer while receiving real-time visual feedback. (**b**) Participants were trained to reach six levels of effort (0, 30, 39, 48, 57, 66% MVC) which were set depending on their individually calibrated MVC and indicated by a cue on each trial. (**c**) Trial outline for the Main task. Participants had to decide whether they first wanted to see the effort level required or the rewards on offer in an ‘order selection’ task. The chosen option was highlighted by a green frame. Following this, participants received a variable offer of an effort/reward combination (ranging between 5 × 5 effort and reward levels), with effort and reward attributes presented successively, according to their choice. The example in (**c**) is of a trial where they chose effort information first. They then decided whether the credits were worth the effort, i.e. whether they wanted to accept or reject the offer. If participants accepted the offer, they had to squeeze above the required effort level for a minimum of 3 s in order to receive the offered credits while receiving real-time visual feedback on their force. Subsequently, they received feedback on the credits earned on that trial. If the offer was rejected, participants rested for the same period of time and received “1 credit”. Note that only in a random 50% of the trials were participants required to squeeze above the effort level if they accepted the offer or to rest if they rejected the offer. Dots represent blank screens.

In this study, we aimed to test whether people have a preference to seek out reward versus effort information first, i.e. to test which of the information they choose to see first on a given trial and choose more frequently to see first overall, and whether any such preference is associated with their willingness to exert effort. For this purpose, we developed a new physical effort-based decision-making task where people made two decisions, one about what information they wanted to receive first, and a second about whether they were willing to exert effort for reward (Fig. 1). On each trial, participants made a choice between two options: a ‘work’ offer, requiring an amount of physical grip force (between 30 and 66% of their maximum voluntary contraction [MVC]) to be exerted in order to obtain rewards (2–10 credits), or a ‘rest’ offer which required no effort for a low reward (1 credit). Prior to making that choice participants made an additional decision between two cues representing seeing the effort or reward information first. Once chosen these cues would reveal the required grip force and offered credits of the work offer on that trial in the order specified (i.e. effort first, reward second if the effort cue was chosen). Thus, participants would receive the same work offers and information on a trial but were able to choose which information they would see first. In addition, participants completed questionnaires of self-reported fatigue and weekly physical activity using established questionnaire measures. Using this design we could test the hypotheses that when people have a preference to seek effort information first they would be less willing to exert effort for reward. Moreover, we could test whether this effect was associated with fatigue and exercise in everyday life.

## Results

### No overall preference for effort or reward information, but significant variability

**Figure 2:**
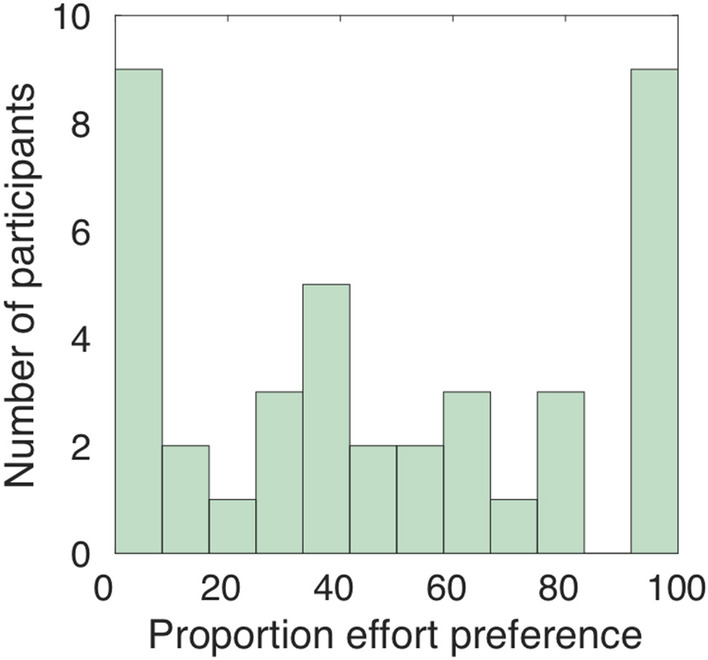
**General preference.** Depicted is the percentage of trials across the experiment in which a participant chose to first see the effort information of the work offer on that trial. The histogram illustrates variability in choosing effort or reward information first, both across and within participants.

First, we tested whether people have an overall preference for seeking effort or reward information first when making effort-based decisions. For each participant we calculated the percentage of trials on which they had chosen to see effort versus reward information first, and compared them with a non-parametric Wilcoxon signed-rank test. Overall, there was no statistically significant difference for choosing effort or reward information first before option selection, *Z* = − 0.440, two-tailed *p* = 0.660, indicating that there was no evidence that people overall had a preference to know effort or reward information first. However, there was considerable variability between people (Fig. 2), with preferences ranging fully between 100% effort or reward information first. Moreover, 25 of the participants showed variability in their preference, selecting neither effort nor reward information first on every trial. Thus, there is significant variance, both intra-individually and inter-individually, in participants’ preferences to seek effort versus reward information first. Note that because the location (left/right) of the two options representing the effort and reward information was randomised across trials, and because there was considerable within-subject variability in choosing the left or the right option during the ‘order selection’ task (left option chosen on *M* = 52.167% of trials [*SD* = 6.887]), the observed preferences cannot simply be due to a potential preference to always select the left or the right option on the screen.

### Choices to seek effort information first, predict subsequent decisions to exert effort

**Figure 3:**
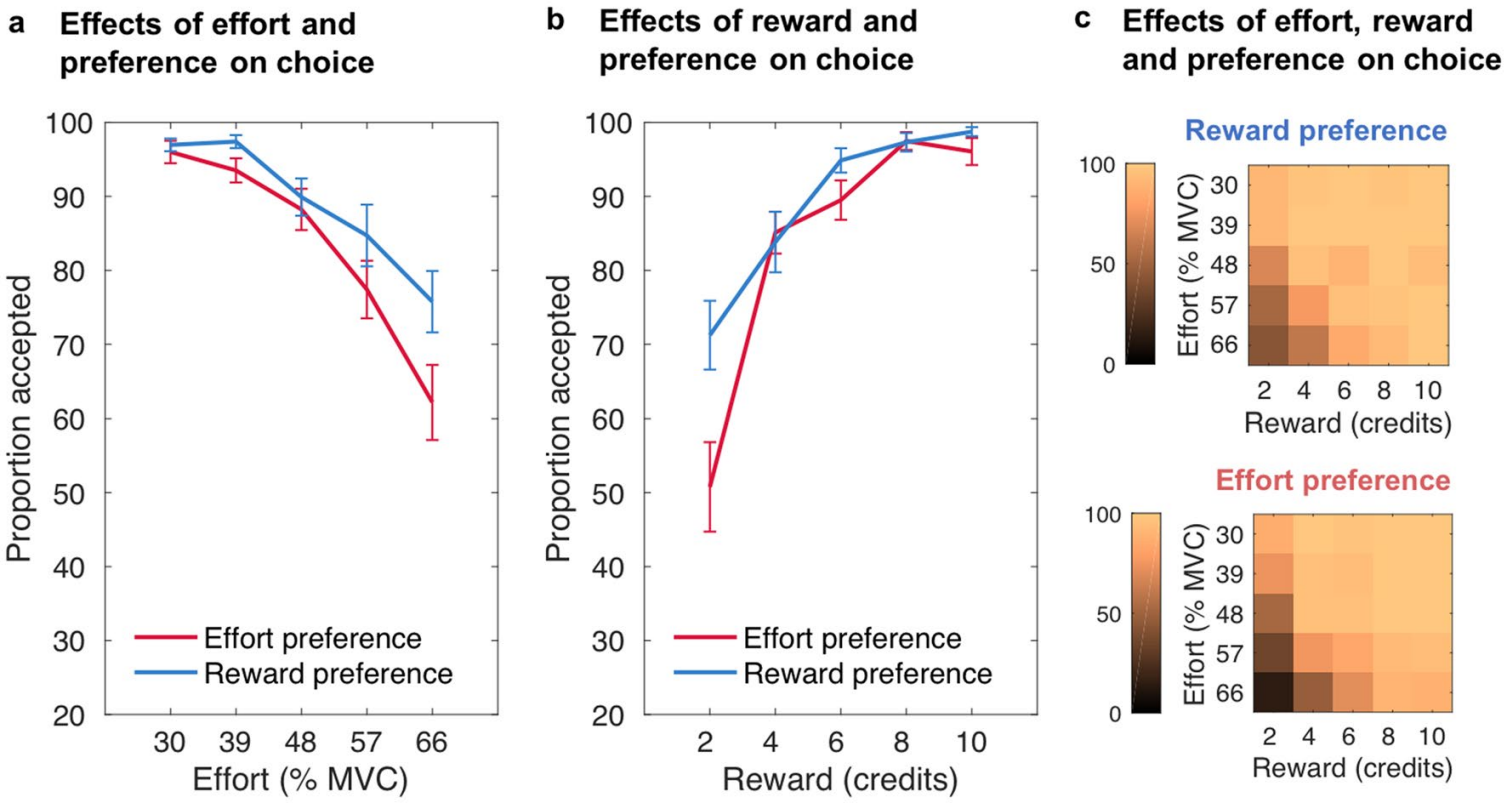
**The willingness to exert effort on a trial depends on participants’ preferences for seeking effort information first on a trial**. Mean proportion of accepted work offers as a function of preference for effort or reward information first on a given trial, dependent on (**a**) the effort level, (**b**) the reward level, and (**c**) the effort and reward levels of the work offer. Reward preference (blue) depicts choice behaviour on trials on which participants chose to first see reward information, while effort preference (red) depicts choice behaviour on trials on which participants chose to first see effort information. There was an interaction between preference and effort level on choice, indicating a reduced willingness to exert higher levels of effort for rewards if participants had chosen to see the effort required before the reward on offer. Error bars represent standard errors of the means.

Next, we tested the hypothesis that having a preference for seeing effort information first on a given trial would be associated with a reduced willingness to exert effort^43^. To test this a generalised linear mixed-effects model (GLMM) on choices to work or rest on trials *n* was run with effort level, reward level and information seeking preference on trials *n* as well as all interactions as predictors (see also Supplementary Table S1 online). This revealed a significant main effect of preference, χ^2^(1) = 5.841, *p* = 0.016, and a significant interaction of preference and effort level, χ^2^(1) = 6.729, *p* = 0.009, showing that choosing effort information first was associated with an increased likelihood of rejecting offers, in particular those higher in effort as confirmed by a steeper slope of the effort predictor for effort versus reward preferences, *Z* = -2.975, *p* = 0.003 (see also Fig. 3a). There were also significant main effects and a significant interaction of effort level and reward level (effort: χ^2^(1) = 262.197, *p* < 0.001; reward: χ^2^(1) = 288.486, *p* < 0.001; effort × reward: χ^2^(1) = 4.374, *p* = 0.036), demonstrating that participants considered both effort and reward information when deciding whether the reward on offer was worth the required effort, rejecting higher effort and lower reward offers. All other interactions were not significant (preference × reward: χ^2^(1) = 0.215, *p* = 0.643; preference × effort × reward: χ^2^(1) = 2.413, *p* = 0.120; Fig. 3b and c), although Fig. 3b, for example, might suggest that reward preference may have been associated with a somewhat increased acceptance of low reward offers. Notably, the main effect of trialwise preference remained marginally significant when an additional variable representing the general preference (see below) was additionally included in the model (*p* = 0.06).

### General preferences for seeking effort information associated with reduced willingness to exert effort

**Figure 4:**
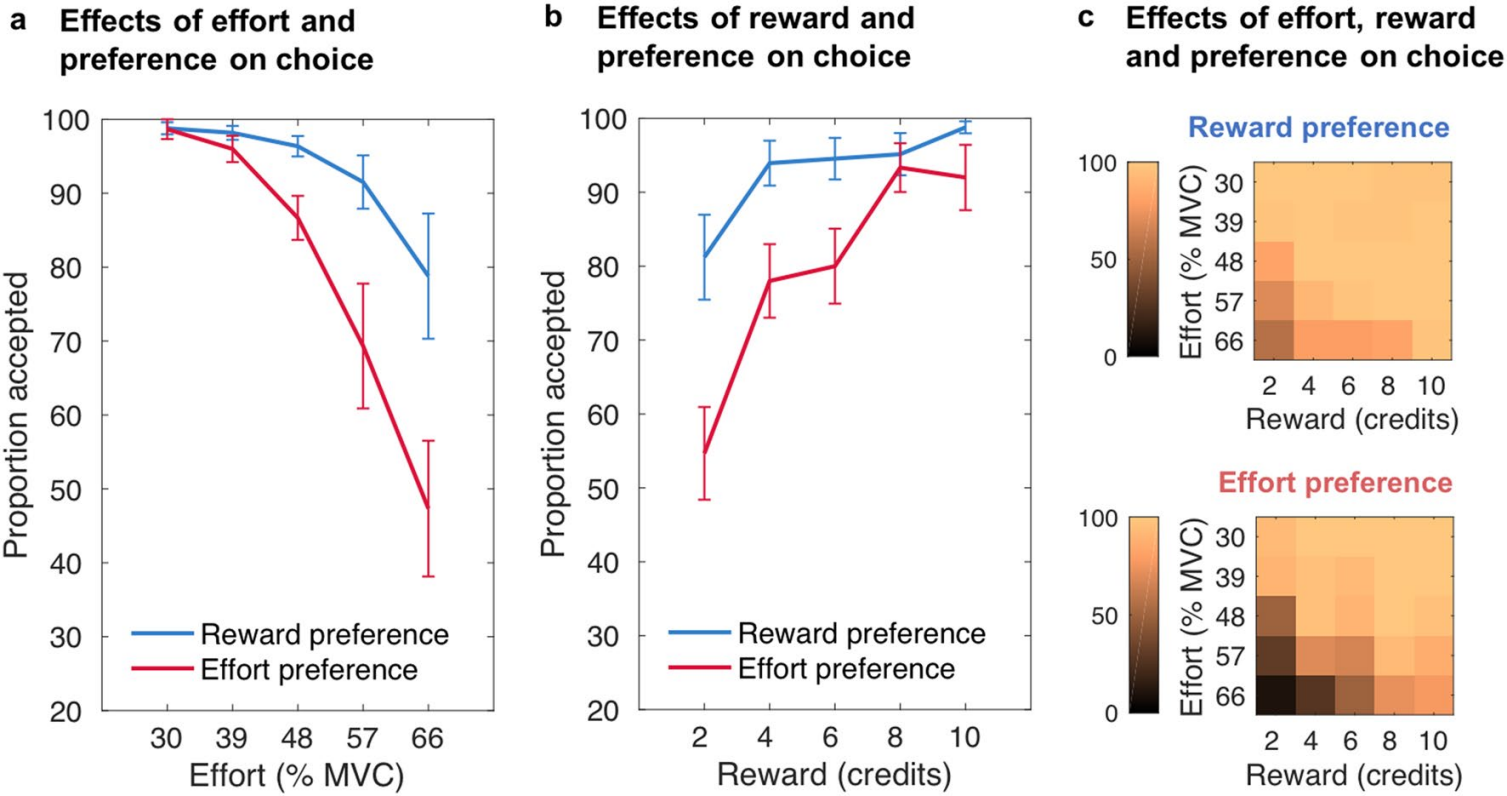
**The willingness to exert effort for reward varies with general preferences for seeking effort or reward information first.** Mean proportion of accepted work offers as a function of general preference (proportion of trials choosing effort or reward first), dependent on (**a**) the effort level, (**b**) the reward level, and (**c**) the effort and reward levels of the work offer. Although statistical analyses used a continuous variable, for illustration purposes here reward preference reflects the participants who chose to first see reward information in at least 80% of all trials, while effort preference depicts choice behaviour from participants who chose to first see effort information in at least 80% of trials. There was an interaction between people’s information seeking preference and willingness to exert effort, with people favouring seeking effort information first less willing to exert higher levels of effort for rewards than people favouring seeing reward information first. Error bars represent standard errors of the means.

Next we tested the hypothesis that stable, general preferences for seeking effort or reward information first would be associated with a reduced willingness to exert effort. Thus, to examine whether the variability between participants (Fig. 2) in general preferences for seeking effort or reward information first was predictive of the willingness to exert effort for reward, an additional GLMM including the same predictors as above was constructed, but including the proportion of trials across the task on which effort information was chosen first (general preference) rather than trial-by-trial preferences (see also Supplementary Table S2 online). In line with the hypothesis, the more often a participant chose to see effort information first, the more likely they were to reject work offers, in particular those higher in effort (general preference: χ^2^(1) = 6.464, *p* = 0.011; general preference × effort: χ^2^(1) = 15.472, *p* < 0.001; Fig. 4a). In addition, the higher the effort level and the lower the reward level the more likely participants were to reject the offer (effort: χ^2^(1) = 249.656, *p* < 0.001; reward: χ^2^(1) = 288.806, *p* < 0.001; effort × reward: χ^2^(1) = 5.058, *p* = 0.025). All other interactions were not significant (general preference × reward: χ^2^(1) = 0.205, *p* = 0.651; general preference × effort × reward: χ^2^(1) = 1.992, *p* = 0.158; Fig. 4b and c). Notably, the main effect of general preference remained marginally significant when an additional variable representing trialwise preference was additionally included in the model (*p* = 0.05).

To support these findings, we fitted a well-established parabolic effort-discounting model to participants’ effort-based decisions in the task. The parameter dictating how much an individual discounts rewards by effort (*k*) was correlated with each participant’s general preference using Spearman’s rank correlation coefficient. The results revealed a significant positive relationship between a person’s tendency to choose to see effort information first and this person’s tendency to reject offers that would require the exertion of higher effort, *r*_s_ = 0.375, two-tailed *p* = 0.017.

### Preference for effort information seeking associated with slower reaction times

**Figure 5:**
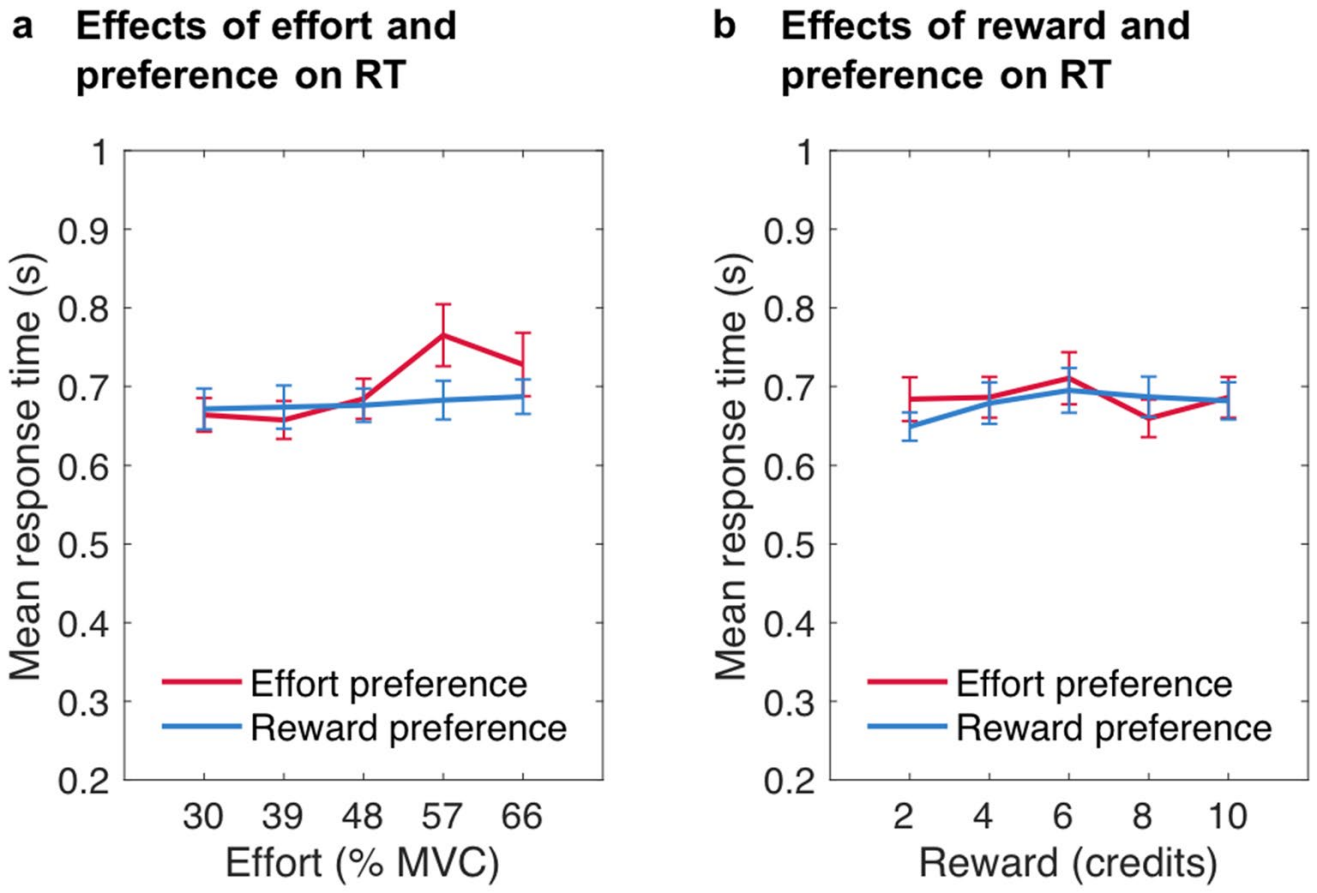
**Response times for accepted offers dependent on participants’ preferences on a trial.** Mean response times (RTs) in seconds dependent on (**a**) the effort level and (**b**) the reward level of the work offer, as a function of preference on a given trial. Reward preference depicts response times on trials on which participants chose to first see reward information, while effort preference depicts response times on trials on which participants chose to first see effort information. Error bars represent standard errors of the means.

To support these analyses and further investigate how preferences for effort or reward information may affect choices, a separate linear mixed-effects model (LMM) on participants’ reaction times (RTs) in trials in which they had accepted the work offer was run, with effort level, reward level and preference for information on trials *n* as well as all interactions as predictors (see also Supplementary Table S3 online). The analysis was restricted to trials where participants chose to work, as there were not enough trials rejected at some combinations of effort and reward levels. Participants were slower to accept offers high in effort compared to offers low in effort, χ^2^(1) = 29.043, *p* < 0.001. There was also a significant main effect of reward, χ^2^(1) = 17.339, *p* < 0.001. The main effect of preference was not significant, χ^2^(1) = 0.720, *p* = 0.396, while the interaction of preference and effort level was close to being significant, χ^2^(1) = 3.738, *p* = 0.053, with the slope of the effort predictor however being higher for effort versus reward preferences, *t* = 1.970, *p* = 0.049 (see also Fig. 5a). The other interactions were not significant (effort × reward: χ^2^(1) = 1.237, *p* = 0.266; preference × reward: χ^2^(1) = 0.063, *p* = 0.801; preference × effort × reward: χ^2^(1) = 0.430, *p* = 0.512; Fig. 5).

**Figure 6:**
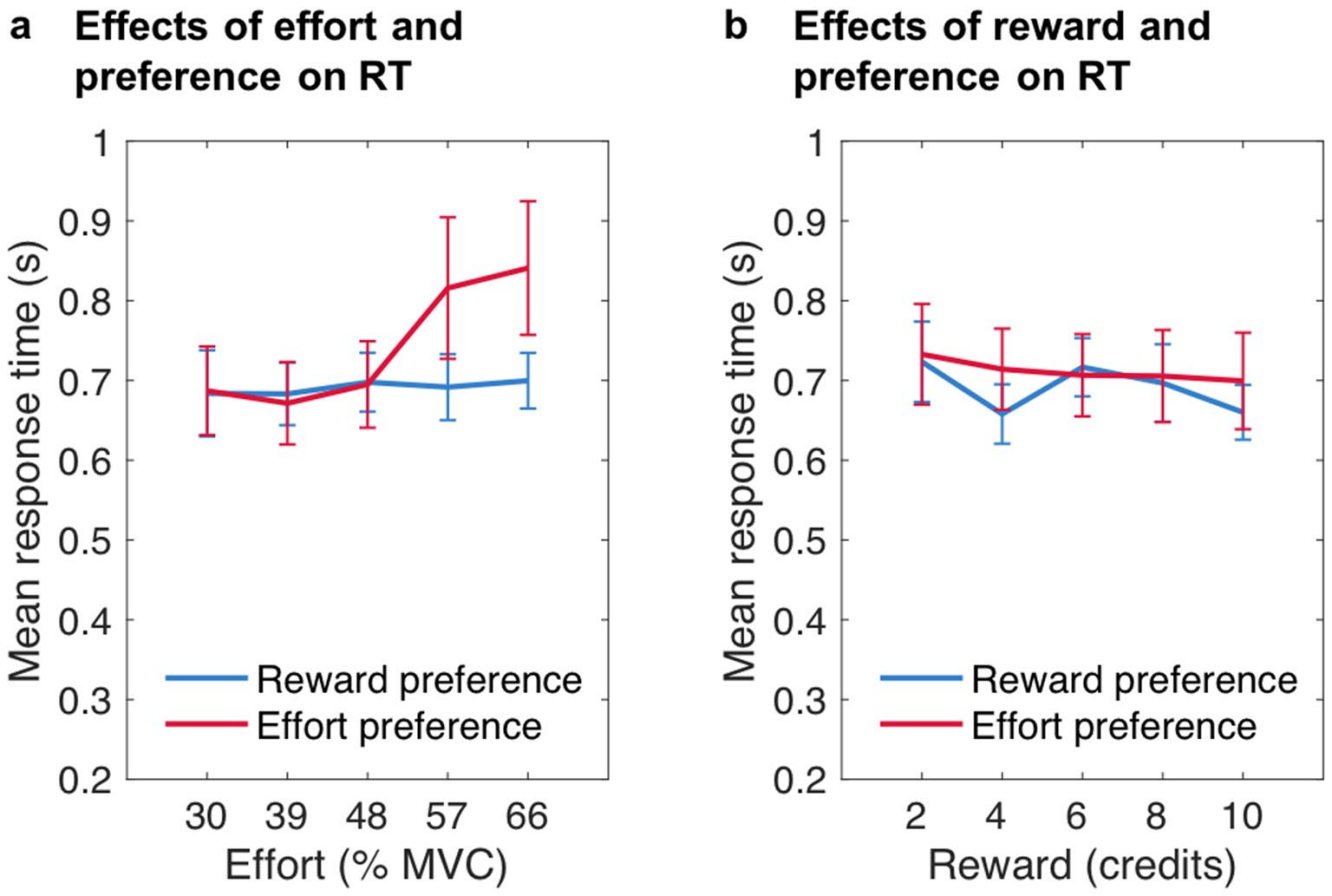
**Response times for accepted offers dependent on participants’ general preferences.** Mean response times (RTs) in seconds dependent on (**a**) the effort level and (**b**) the reward level of the work offer, as a function of general preference. For illustration, reward preference depicts response times from participants who chose to first see reward information in at least 80% of all trials, while effort preference depicts response times from those participants who chose to first see effort information in at least 80% of trials. Note that here the slope of the effort predictor was significantly higher for effort preference compared to reward preference, *t* = 3.035, *p* = .003. Error bars represent standard errors of the means.

In an additional LMM in which preference was exchanged with general preference as predictor (see also Supplementary Table S4 online), when participants selected effort first in a high proportion of trials they were particularly slow in accepting higher effort offers (general effort preference × effort: χ^2^(1) = 8.273, *p* = 0.004; Fig. 6a). There were also significant main effects of effort, χ^2^(1) = 29.632, *p* < 0.001, and reward, χ^2^(1) = 18.385, *p* < 0.001, but none of the other effects significantly predicted RTs when accepting work offers (effort × reward: χ^2^(1) = 1.462, *p* = 0.227; general effort preference: χ^2^(1) = 0.355, *p* = 0.551; general effort preference × reward: χ^2^(1) = 0.191, *p* = 0.662; general effort preference × effort × reward: χ^2^(1) = 0.079, *p* = 0.778; Fig. 6). The finding that people who tended to prioritise effort information first were slower at accepting work offers that required exertion of higher effort than people who usually preferred to see reward information first provides further evidence that effort preference is associated with a bias against high effort options.

### Physical effort sensitivity is related to general preferences for seeking effort information first

**Figure 7:**
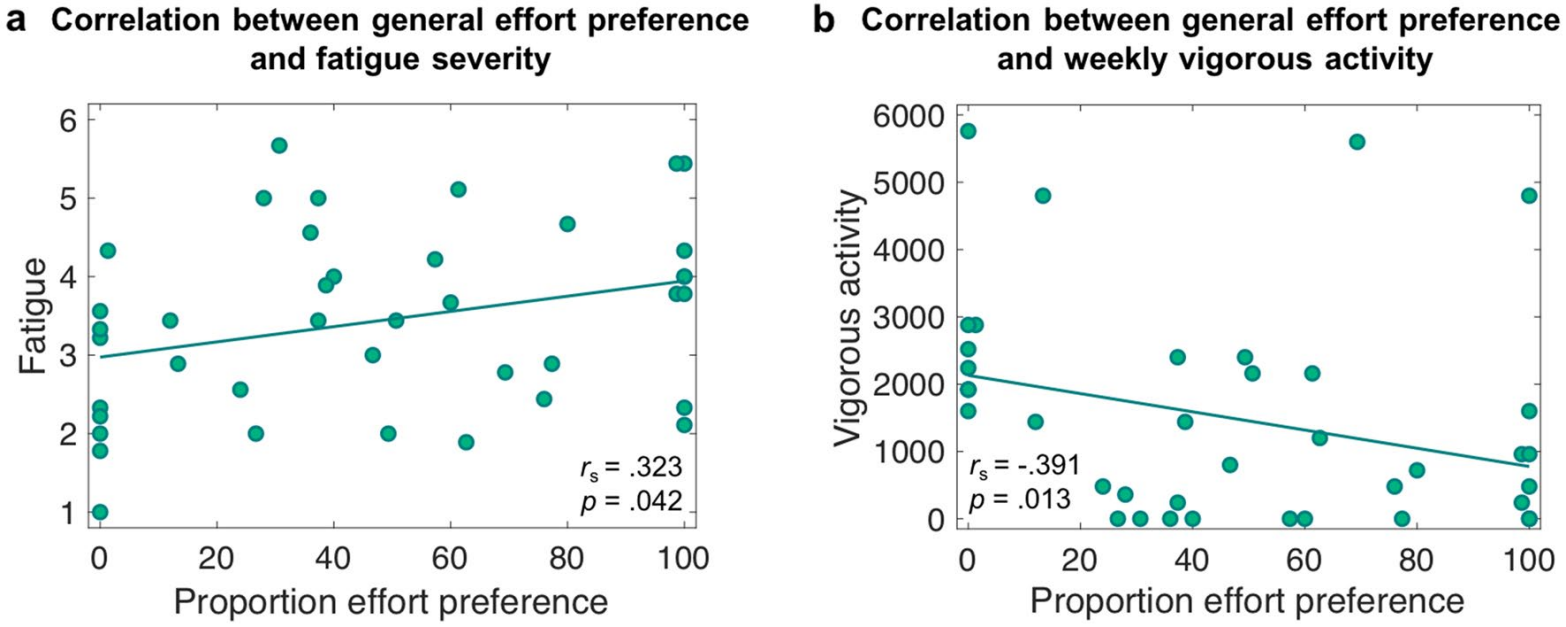
**Correlation between general effort preference and effort sensitivity.** (**a**) Correlation between the percentage of trials in which a participant chose to first see the effort information and self-reported fatigue (FSS score on a scale between 1 and 7). (**b**) Correlation between the percentage of trials in which a participant chose to first see the effort information and the intensity of vigorous activity undertaken, as assessed with the IPAQ. Vigorous activity is reported in MET-minutes per week. Figures show that a stronger preference for seeking effort information first is associated with higher levels of fatigue, and with reductions in vigorous physical activity in everyday life.

Next we tested the hypothesis that a preference for seeking effort information first, which is associated with a reduced willingness to exert effort, might be related to heightened levels of fatigue and reduced exercise in everyday life. To test this, we correlated self-reported fatigue (Fatigue Severity Scale [FSS] score) and weekly time spent with vigorous-intensity activities (International Physical Activity Questionnaire [IPAQ] score) with people’s general preference for seeking effort or reward information first. As the data was not normally distributed, Spearman’s rank correlation coefficient was computed. Two-tailed *p*-values are reported. General effort preference was associated with higher self-reported fatigue severity, *r*_s_ = 0.323, *p* = 0.042 (Fig. 7a). In addition, there was a significant negative correlation between effort preference and a person’s vigorous physical activity, *r*_s_ = − 0.391, *p* = 0.013 (Fig. 7b). There were no significant correlations between general effort preference and weekly time spent with moderate physical activity, *r*_s_ = − 0.180, *p* = 0.267, or between general effort preference and weekly time spent walking, *r*_s_ = − 0.189, *p* = 0.242, in the IPAQ. These results indicate that people who become more easily fatigued with exercise, or who are more impacted by fatigue, and who do less vigorous intensity exercise tend to prioritise effort information more than people who are less susceptible to fatigue or who do more vigorous intensity exercise per week.

## Discussion

In this study, we investigated whether people have a systematic preference for seeking information about the rewards or the effort associated with behavioural options first, and whether this potential preference is associated with people’s tendency to be averse to exerting effort for reward. Before making decisions on whether to accept or reject a work offer, participants were required to decide whether they wanted to look at the effort on offer first, or the reward on offer first. Results revealed that there was no overall preference for effort or reward information, but as hypothesised, the information which was chosen to be seen first predicted subsequent choice behaviour. Specifically, a general preference for seeking effort information first, and trial-by-trial choices of effort information first, were associated with a reduced willingness to exert higher effort. The same patterns were evident in people’s RTs, with slower responding to accept higher effort offers on trials where effort was chosen first, and when people had a preference for seeking effort information first overall. Lastly, a preference for seeking effort information first was associated with a higher impact of fatigue and reduced vigorous exercise, in daily life. These results highlight the importance of how people seek information about effort and reward, for influencing people’s willingness to exert effort and levels of daily exertion.

These findings extend recent evidence that attending to cost information first (versus last) is associated with reduced motivation to exert cognitive effort^43^. Here, this effect was demonstrated where a preference to seek effort information first was associated not only with a decreased willingness to exert high physical effort for reward but also with increased response times when highly effortful options where nevertheless chosen, likely indicative of a shift in the strength of preference^44^. Notably, these effects were observed even though people received the same information on a trial regardless of what they sought first, and unlike in previous studies on cognitive effort in which both presentation order and presentation duration of effort and reward information were manipulated simultaneously^43^. While both effort and reward information were nonetheless taken into account when deciding whether exerting effort for reward was worth it, choosing to see effort information first appears to have placed a higher weight on the effort associated with the work offer and thereby have increased effort discounting during subsequent decision-making.

What mechanisms might underlie this process? Speculatively, it has been suggested that the order in which information enters the prefrontal cortex influences how much weight is put on that information in subsequent behaviour. Early information is prioritised, and thus influences behaviour to a greater degree^43^. Given the known role of the prefrontal cortex in effort-based decisions^7^, and in particular those in very similar paradigms to this study, such a mechanism seems highly plausible. Similarly, some findings suggest that the order in which information is attended may bias choices, by influencing how evidence is accumulated towards making a choice^19,37^. Thus, it may be possible that earlier entry of effort cost information, in the absence of knowing the reward, biases the accumulation of evidence towards rejecting a behaviour.

Although this might explain how effort prioritisation might change the willingness to exert effort, it cannot explain people’s initial preferences to seek effort or reward information first. Research on information seeking has suggested that there are a number of different motivations and biases that influence what information we seek or avoid^21^. A recent account characterises three potential utilities that define whether someone seeks or avoids information: instrumental (will the knowledge help, or hinder decisions that increase rewards or avoid costs), affect (will the information induce positive or negative emotions) and cognition (will information improve the ability to understand and predict my environment)^21^. Although it is clear that the information seeking in this experiment could be seen as influencing the potential for obtaining rewards and therefore be linked to instrumental utility, there are several features which suggest a need for further considerations in such theories. Specifically, information seeking is typically studied in paradigms where participants can either receive information or not. In this study, participants were always told about both variables guiding their decisions, both effort and reward, on every trial. All participants chose was which information they saw first. However, current accounts of information seeking have not thoroughly discussed how information seeking might be biased or differ when the only choice is the temporal order of information. Our results suggest that there is a stable link between preferring to know effort information first and being less motivated. Unfortunately in this design it is not possible to determine the causal direction of these effects, whether feeling demotivated or fatigued leads one to seek effort information first, or whether seeking effort information first reduces motivation. However, our results do point to the need for theories of both information seeking and motivation to be brought together to understand how information seeking and the willingness to exert effort interact^45^.

Notably, preferences for effort or reward information were associated with interindividual differences. The more participants had experienced an impact of fatigue in their daily life in the last week, and the less vigorous activity they were doing on a weekly basis, the more pronounced was their preference for effort information. These results stress that the preferences captured in this task are related to, and relevant for, everyday choices and behaviour and may be a somewhat stable individual difference. With evidence suggesting that cognitive and physical effort may share mechanisms and often show similar patterns of effects^7^, our results raise the possibility that this stable preference for seeking effort information first may occur for both cognitive and physical effort^43^. Speculatively at the neural level, differential attention to and amplification of effort or reward information might be associated with individual dopamine availability in the striatum, as suggested by recent studies on people’s motivation to expend cognitive effort^46,47^. Such a link between effort information seeking and effort-based choice could constitute an adaptive mechanism^34,48^ that simplifies decision-making and prevents exhaustion. However, it may come at the cost of leading to irrational choices and maladaptive, persistent effort avoidance in some people^48,49^.

Our results are in line with recent accounts and findings suggesting that fatigue may increase sensitivity to effort costs during effort-based choices, and bias decisions about whether to act^5,28,50^. That is, as fatigue increases it may direct attention towards effort information^19,30,31,33,34^, which may in turn lead to an increased likelihood of avoiding effort. The relationship between self-reported fatigue, reduced vigorous exercise and prioritising of effort information in this study supports this notion. Theories have suggested that both state and trait fatigue might be associated with an increased focus on internal bodily signals^5,25,30^ and that attended information may receive greater weight in the decision-making process^19,33^. For instance, during demanding physical exercise, attention may become more focused on physiological sensations^51,52^. Our results suggest that people who may be more focused on their internal states and who undertake less vigorous exercise may also have a preference to first learn about how effortful a behaviour is, before finding out how rewarding it will be. As noted above, the causal direction of this effect is unclear. It remains to be disentangled in future research whether heightened sensitivity to effort and thus increased subjective saliency of effort information indeed results in increased attention to effort and thus in effort avoidance, or to what exent attention to effort information may increase the subjective perception of exerted effort. Nevertheless, the present work highlights that people’s attention to physiological sensations, reduced motivation, and seeking of information may be closely linked together. Thus, it may be fruitful for future work to focus on reducing the impact of fatigue, and motivational impairments such as apathy, to consider how people seek information that influences motivation.

Previous work suggested that optimised work-rest schedules could help ameliorate persistent forms of fatigue^25,53^. The current findings might aid the development of new strategies for people to overcome maladaptive choice biases and maladaptive forms of behaviour, for example by carefully considering the presentation order of choice-relevant information or by systematically directing attention towards the rewards on offer. For instance, in recent research on depression, a disorder associated with heightened levels of fatigue and amotivation^10,54,55^, attentional bias modification was found to have positive effects on residual symptoms by leading to more adaptive emotion perception and emotion regulation through the reduction of negative attentional biases^56–58^. Potentially, similar trainings may prove useful for modifying effort (or reward) biases. This may in turn have direct positive effects on feelings of fatigue, or it may help modify maladaptive forms of behaviour. The latter option is perhaps more likely and may indeed be efficacious as previous work suggested that physical exercise, when done in appropriate form, intensity, duration and intervals, could help improve or prevent fatigue in the long run, potentially also in pathological conditions^25,53,59^. Although it should be noted that the effect of exercise on fatigue in some clinical disorders, such as Parkinson’s disease, is less clear as yet^60–62^.

There are limitations to the design of this study, that temper some stronger conclusions, particularly about the causality of how information seeking and motivation intersect. As discussed earlier, we note that our study design does not allow for conclusions about the causal direction of the observed effects and thus the extent to which information seeking preferences affect motivation or if motivation affects information seeking preferences. Moreover, the precise mechanism by which a heightened focus on effort may be associated with fatigue and physical inactivity is interesting and offers potentially important impact. However, our measure of physical inactivity is self-report, rather than a real-world measurement, and these effects need to be replicated and the causal direction to be understood before it will be possible to develop a full framework of why people show reduced activity in their day-to-day lives. Furthermore, as our task examined physical effort only, but the fatigue severity scale does not distinguish between cognitive and physical effort, we are limited in whether our effects will generalise to cognitive effort and mental fatigue. Yet it is important to note that the effect of effort information first on decisions to exert effort was in line with that of Vassena et al.^43^, who used a cognitive effort task, and thus it appears likely that our effect would translate across different types of effort and fatigue. Lastly, we note that the effects of the offered reward and the required effort associated with the work options seem larger than the effects of people’s information seeking preferences. While previous work has shown that the effort and reward levels used in this study have considerable effects on decisions to exert effort for reward but are also somewhat malleable for instance when participants become fatigued^28^, our main goal was to highlight that these effects can also be malleable by the order in which information is chosen. We refrained from reporting effect size estimates as currently there does not seem to be a generally accepted or preferred way to compute standardised effect sizes for mixed-effects model analyses such as the ones reported here, with existing approaches to compute effect size estimates for rather complex mixed-effects models typically entailing some theoretical and practical challenges^43,63^, yet promising new approaches are being developed^63^. The limitations outlined here can be addressed in future research to develop a rich framework for how information seeking and motivation intersect.

In summary, this study revealed that although people overall do not have a preference for seeking effort or reward information first, those who do typically seek effort information—or when people choose effort first at a particular moment in time—show greater effort discounting. That is, having chosen to know how effortful a behaviour is first, the cost is inflated and people’s willingness to exert effort for reward reduced. This pattern was present in both people’s choices and also reflected in slower RTs to accept a high effort offer having chosen to know the effort first. Moreover, we showed that people who do prefer to seek effort information first, reported higher levels of fatigue in their everyday life and spent less time engaged in vigorous daily activities. As such, how people seek information that underpins their willingness to exert themselves appears crucial for everyday levels of daily activity. A better understanding of such choice biases and how they might develop into maladaptive forms (too little or too much focus on effort or rewards) may help prevent and improve abnormal decision-making, maladaptive behaviour and persistent fatigue in everyday life.

## Methods

### Participants

Forty healthy adult participants (24 females) with a mean age of 25.10 years (*SD* = 5.42; range 18–36) and with no history of neurological or psychiatric illness were recruited through the Oxford Psychology Research participant recruitment scheme and respective online bulletin boards. The sample size was selected based on related previous studies assessing effort-based decisions and the subjective valuation of effort costs^7,28,43^. The research was approved by the South Central—Oxford A Research Ethics Committee (18/SC/0448), and written informed consent was obtained from all participants prior to the experiment in accordance with the ethical standards laid down in the Code of Ethics of the World Medical Association (Declaration of Helsinki).

### Experimental design and procedure

The overall aim of the experiment was to examine how seeking reward or effort information first might bias people’s decisions of whether to exert effort for reward. To thoroughly address this question we aimed to examine (1) whether there was an overall bias for people to prefer effort or reward information first, (2) whether a person’s overall tendency to choose effort or reward information first was associated with the willingness to exert effort for reward, (3) whether on specific trials where people chose effort or reward first, people were more or less willing to exert effort for reward and (4) whether people’s tendency to seek effort or reward information first was associated with everyday levels of fatigue and exercise. To do this, we created a two-step, effort-seeking-effort-based decision-making task, adapting existing physical effort experiments^7,14,28^, where participants could choose whether to know the effort or reward of an offer first, before subsequently deciding whether to exert that effort.

The experimental procedure consisted of three parts (Fig. 1): (1) a *Calibration* phase to account for individual differences in grip strength, (2) a *Training* phase in which participants familiarised themselves with the effort levels used in this task, and (3) the *Main task*. In the Main task, participants decided on every trial whether they thought the credits on offer were worth the force level required to obtain them, with the total number of credits collected throughout the task determining a bonus (range: £0–£4) on top of their basis payment (£8).

During *Calibration* (Fig. 1a), each participant’s MVC was measured by squeezing a hand-held dynamometer on three consecutive trials with their dominant hand. Participants were required to apply as much force as possible on each trial, and they received strong verbal encouragement while squeezing. During each attempt, a bar presented on the screen provided real-time feedback of the force being generated. In the second and third attempts, a benchmark representing 105% and 110%, respectively, of the previous best attempt was used to encourage participants to improve on their score. The maximum level of force generated throughout the three attempts was used as MVC.

In the *Training* phase (Fig. 1b), participants practised reaching each of six effort levels (0, 30, 39, 48, 57, and 66% of each participant’s MVC). The trial was successful only when the force generated by the participant exceeded the required level for a sum total of at least 3 s in a five-second window. Practice of each effort level was repeated three times, resulting in 18 practice trials in total. Each trial commenced with a cue in the form of a bar, with a yellow line superimposed on the bar and a red filling indicating the upcoming effort level. To make sure that participants carefully and successfully completed this training, they were awarded one credit for each successful squeeze, while they received zero credits for a failure.

The *Main task* (Fig. 1c) consisted of 75 trials, each requiring participants to make two decisions. In the first decision phase, participants were asked to decide whether they first wanted to receive information on the effort required or whether they first wanted to see information on the rewards on offer on that trial. In the second decision phase, participants had to decide whether they find the rewards on offer are worth the required effort. On each trial, one of five different effort levels that corresponded to 30, 39, 48, 57, and 66% of each participant’s MVC and one of five different reward levels (2, 4, 6, 8, 10 credits) was presented. Effort and reward levels were varied independently and presented in a pseudo-random order to ensure that each effort/reward combination was distributed evenly across the task.

For the choice about what information to see first at the start of the trial, effort information was represented by a vertical bar on one side of the screen, and reward information was represented by the word “credits” on the other side of the screen. The location (left/right) of the two options representing the effort and reward information was randomised across trials to ensure that participants’ potential preferences could be dissociated from a potential preference to always select the left or the right option. Note, at this point the effort and reward information was hidden and if chosen these cues simply indicated what would be presented first. Participants had to respond within 3.5 s, pressing keys “b” and “n” on a keyboard with their index and middle finger, respectively, to select the left or the right option presented on the screen. Subsequently, according to the participant’s choice, effort and reward information were presented successively, each for a duration of 2 s, separated by a blank screen presented for 1 s. Presenting both pieces of information for the same duration ensured that presentation order and presentation duration were not confounded. Information on the effort level was indicated by a yellow line superimposed on the bar and a red filling, while reward information was numerically displayed (number of credits). Participants then had to decide whether to reject the offer and rest for a low reward (1 credit) or whether to accept the offer and work for a higher reward. Again, the location (left versus right side of the screen) of the work (“Accept”) and rest (“Reject”) option was randomised across trials. Responses had to be made within 2.5 s, pressing keys “b” and “n”, respectively, to select the left or the right option presented on the screen.

If participants chose to work, they were required to exert the required force on the dynamometer for at least 3 out of 5 s in order to receive the credits associated with the work offer. For this purpose, participants were presented with a vertical bar that provided them with real-time feedback on their force. The target effort level was indicated by a yellow line superimposed on the bar. If participants preferred to rest, the bar was presented for the same duration but with the yellow line displayed at the bottom of the bar. Following this, participants received feedback regarding their success or failure on that trial. The choice period in the following trial was separated from the outcome period in the preceding trial by an intertrial interval of 1 s. Note that if participants did not make a decision on their order preference for effort and reward information within the maximum response time given (first decision), they were allowed one second attempt. If they again failed to give a response, the remainder of the trial was skipped, and participants had to rest for 5 s without receiving any credits. However, the latter of these cases never occurred.

To minimise any of the effects of fatigability on people’s willingness to exert effort, and to maximise the number of trials performed by participants, only on a pseudo-randomly selected 50% of offers were participants actually required to exert effort. In the other cases, a screen with the text “Next trial…” was presented instead of the work or rest screen and the outcome screen for 1.5 s before the intertrial interval. Whilst participants were informed about this, they were instructed to always make their decisions as if they would have to squeeze if they chose the work option. Furthermore, the main task included two breaks, i.e., was split up into three blocks, and participants were free to decide when to continue with the task. The sequence of effort/reward combinations and the structure of the task were identical across participants to ensure that any potential differences in behaviour could be attributed to individual characteristics. Before the actual start of the main task, participants completed three practice trials to familiarise themselves with the task.

### Apparatus

The experiment was conducted in a laboratory room with only the participant and the experimenter present. Stimuli presentation and response collection were implemented using custom code in Matlab (The MathWorks, Inc., USA) and Psychophysics Toolbox extensions^64^, controlled by a PC running the Windows operating system. To examine preferences for effort and reward information and their interaction with the willingness to exert effort, we developed a physical effort-based decision-making task, in which effort was operationalised as the amount of force exerted on a handheld dynamometer (TSD121B-MRI; BIOPAC Systems, Inc., USA). This allowed us to systematically set different, individualised effort levels.

### Self-report questionnaires

Following completion of the task, participants were asked to fill out questionnaires assessing fatigue and physical activity and to provide some demographic information. In accordance with ethical guidelines and to increase the likelihood of participants responding honestly, they were free to skip individual questions if they felt uncomfortable answering them. Inspection of the data however indicated that every participant completed every question.

Fatigue was assessed using the Fatigue Severity Scale (FSS)^27^. The FSS is a nine-item questionnaire assessing the degree to which someone has been susceptible to, and impacted by, fatigue over the past week by providing one score between 1 and 7. The FSS was first developed for use in patients and has subsequently been validated and administered across a wide range of disorders associated with fatigue, as well as in healthy people^65,66^.

To examine a potential relationship between participants’ behaviour on this task and their everyday physical activity, the short version of the International Physical Activity Questionnaire (IPAQ)^67^ was used, which aims to quantify physical activity in adults over the past seven days. Activity is categorized into walking, moderate-intensity activities (for example carrying light loads or bicycling at a regular pace), and vigorous-intensity activities (for example heavy lifting or fast bicycling). Scores are expressed in average MET-minutes/week for each type of activity, with MET (metabolic equivalent of task) representing the energy requirement of an activity as a multiple of the resting metabolic rate. To date, the IPAQ has widely been used across scientific studies and populations.

### Analyses

In the main analyses, choice behaviour was analysed with generalised linear mixed-effects models (GLMMs) using the glmer function from the lme4 package^68^ in R 3.5.2 (R Core Team, 2018), while response times (RTs) were analysed with linear mixed-effects models (LMMs) using the lmer function from the same package with the maximum likelihood estimation method. Using mixed-effects models allowed us to assess all trials and variables of interest within a single model, while accounting for potential variability between participants by including a subject-level random intercept. Choices to work or rest and choices to see effort information or reward information first were coded as binary variables. Trials with missed choices, which occurred very rarely such that no participant had missed more than two trials, were excluded from the analyses. Effort level, reward level and general preference were defined as continuous variables. Effects were tested for statistical significance using a Type II Wald chi-square test, i.e. χ^2^ and *p*-values refer to comparisons between the tested model and the same model without the respective main effect or interaction of interest. Estimated slopes were compared using the emtrends function from the emmeans package^69^.

The inclusion or exclusion of factors was aimed at assuring a good balance between model interpretability, predictive accuracy and model complexity^70,71^. In particular, the mixed-effects models described here were chosen because (1) models with more complex random effects structures did not converge, or they only converged when certain main effects were included as random effects which would however confound interpretations of other effects in our design, (2) they could be used consistently across analyses on choice and reaction time data, facilitating inferences about similarities and differences in results between our various analyses. We however note that mixed-effects models which additionally included a random slope on the main effect of reward per participant were a slightly better fit to the data for the analyses of participants’ choices, albeit not for the analyses of their RTs, according to the Akaike Information Criterion (AIC)^72^ and the Bayesian Information Criterion (BIC)^73^. However, importantly these models showed preference × effort interactions for both choices and RTs (see Supplementary Tables S5–S8 online) as we also found in the models reported in the results. Models with more complex random effects structures did not converge in all analyses. Moreover, regardless of choice of mixed-effects model, we found the same pattern of results in our computational modelling analysis of choice data (see below) and the respective correlation analysis. Thus, our results are consistent and robust to choices of random effects statistical structure.

To specify and quantify individuals’ subjective devaluation of rewards by effort, we additionally fitted a simple discounting model separately to each participant’s choices on whether to exert effort for reward. Based on considerable evidence that rewards are parabolically discounted by physical effort^7,8,14,15,28^, the model assumed that the value of the work offer depends on how rewarding it is, how much effort it requires and how participants subjectively weigh these to guide their choices to work or rest. That is:

1 $${{SV}}_{\left({\rm{t}}\right)}={{R}}_{\left({\rm{t}}\right)}- \left({k }* {{E}}_{\left({\rm{t}}\right)}^{2}\right)$$

where *SV*_(t)_ represents the subjective value of the work option on trial *t*, and *k* represents the subject-specific discount parameter which scales the devaluation of a reward (*R*, reward level 2, 3, 4, 5, or 6) by the effort (*E*, effort level 2, 3, 4, 5, or 6) required to obtain the reward. The higher an individual’s *k* parameter, the steeper an individual’s discount function, i.e. the more this individual’s valuation of rewards is discounted by the effort required to obtain the rewards. To fit the model to the data, a softmax function was used, estimating the probability *P*_(i,t)_ that a participant will choose the work option *i* that has a subjective value *SV* over the rest option that has a value of 1 (one credit, no effort), defined as:

2 $${{P}}_{\left({\rm{i},{t}}\right)}=\frac{{{e}}^{{{SV}}_{({\rm{i},{t}})}*\upbeta }}{{{e}}^{1 *\upbeta }+ {{e}}^{{{SV}}_{({\rm{i},{t}})}*\upbeta }}$$

Since the baseline *SV* was fixed at 1 (one credit, no effort), when the baseline was chosen *P*_(i,t)_ was calculated according to *P*_(i,t)_ = 1−*P*_(i,t)_. Maximum likelihood estimation, using the fminsearch function in Matlab 2017b, was used to minimise the difference between each participant’s actual choices and the model estimates for each trial, i.e. to minimise the negative log-likelihood. The estimates of the discounting parameter *k* and the level of stochasticity in the choices (*ß*) were restricted not to go below 0.0276 (in which case even the combinations of lowest reward and highest effort are always accepted) and 0, respectively. The model was fitted 50 times using different random starting values (using rand) to ensure that the optimisation function had not settled on a local minimum.

## Supplementary Information

Supplementary Tables.

## Data Availability

Source data for the figures as well as the data collected and analysed during this study are available on the Open Science Framework (OSF; https://osf.io/sr97j/; Digital Object Identifier: https://doi.org/10.17605/OSF.IO/SR97J).

## Code availability

Main experimental code and custom Matlab code to implement the computational model are also available on OSF (https://osf.io/sr97j/; Digital Object Identifier: https://doi.org/10.17605/OSF.IO/SR97J).
