# Supplementary material for: Preferences for seeking effort or reward information bias the willingness to work
Source: Sci Rep. 2022 Nov 14;12:19486. doi: 10.1038/s41598-022-21917-7 (PMC9663561; doi:10.1038/s41598-022-21917-7)
Supplement: Supplementary file 1 — Supplementary Tables. [file 41598_2022_21917_MOESM1_ESM.pdf]

Preferences for seeking effort or reward information bias the willingness to work

Tanja Müller, Masud Husain, Matthew A. J. Apps

Supplementary Information

Supplementary Table S1

Analysis of deviance (Type II Wald chi-square test) from the generalised linear mixed-effects model on choice data with trial-by-trial preference as predictor

Predictor	χ^2	df	*p*-value
Preference	5.8405	1	0.015661
Effort	262.1974	1	< 2.2e-16
Reward	288.4857	1	< 2.2e-16
Preference:Effort	6.7292	1	0.009485
Preference:Reward	0.2145	1	0.643296
Effort:Reward	4.3741	1	0.036490
Preference:Effort:Reward	2.4129	1	0.120343

Note. With the choice coded as a binary outcome variable, preference, effort, reward and their interactions were defined as fixed effects. Preference was coded as a binary variable while effort and reward were coded as continuous variables. A subject-level random intercept was included. AIC = 1318.9; BIC = 1373.0

Supplementary Table S2

Analysis of deviance (Type II Wald chi-square test) from the generalised linear mixed-effects model on choice data with general preference as predictor

Predictor	χ^2	df	*p*-value
Preference	6.4636	1	0.01101
Effort	249.6562	1	< 2.2e-16
Reward	288.8055	1	< 2.2e-16
Preference:Effort	15.4723	1	8.372e-05
Preference:Reward	0.2050	1	0.65075
Effort:Reward	5.0578	1	0.02451
Preference:Effort:Reward	1.9924	1	0.15809

Note. With the choice coded as a binary outcome variable, preference, effort, reward and their interactions were defined as fixed effects. All predictors were coded as continuous variables. A subject-level random intercept was included. AIC = 1305.8; BIC = 1359.8

Supplementary Table S3

Analysis of deviance (Type II Wald chi-square test) from the linear mixed-effects model on reaction time data for accepted work offers, with trial-by-trial preference as predictor

Predictor	χ^2	df	*p*-value
Preference	0.7204	1	0.39602
Effort	29.0430	1	7.079e-08
Reward	17.3394	1	3.126e-05
Preference:Effort	3.7379	1	0.05319
Preference:Reward	0.0634	1	0.80117
Effort:Reward	1.2367	1	0.26612
Preference:Effort:Reward	0.4295	1	0.51224

Note. With the reaction time coded as a continuous outcome variable, preference, effort, reward and their interactions were defined as fixed effects. Preference was coded as a binary variable while effort and reward were coded as continuous variables. A subject-level random intercept was included. AIC = -339.4; BIC = -280.8

Supplementary Table S4

Analysis of deviance (Type II Wald chi-square test) from the linear mixed-effects model on reaction time data for accepted work offers, with general preference as predictor

Predictor	χ^2	df	*p*-value
Preference	0.3552	1	0.551168
Effort	29.6318	1	5.224e-08
Reward	18.3854	1	1.804e-05
Preference:Effort	8.2730	1	0.004024
Preference:Reward	0.1911	1	0.662032
Effort:Reward	1.4615	1	0.226696
Preference:Effort:Reward	0.0791	1	0.778487

Note. With the reaction time coded as a continuous outcome variable, preference, effort, reward and their interactions were defined as fixed effects. All predictors were coded as continuous variables. A subject-level random intercept was included. AIC = -342.9; BIC = -284.4

Supplementary Table S5

Analysis of deviance (Type II Wald chi-square test) from the generalised linear mixed-effects model on choice data with trial-by-trial preference as predictor, additionally including a random slope on reward

Predictor	χ^2	df	*p*-value
Preference	6.7161	1	0.009555
Effort	242.7465	1	< 2.2e-16
Reward	59.3277	1	1.335e-14
Preference:Effort	5.5135	1	0.018870
Preference:Reward	2.4271	1	0.119255
Effort:Reward	8.3950	1	0.003763
Preference:Effort:Reward	4.2565	1	0.039100

Note. With the choice coded as a binary outcome variable, preference, effort, reward and their interactions were defined as fixed effects whereby a random slope on the main effect of reward per participant was additionally included. Preference was coded as a binary variable while effort and reward were coded as continuous variables. A subject-level random intercept was included. AIC = 1244.2; BIC = 1310.2

Supplementary Table S6

Analysis of deviance (Type II Wald chi-square test) from the generalised linear mixed-effects model on choice data with general preference as predictor, additionally including a random slope on reward

Predictor	χ^2	df	*p*-value
Preference	5.9857	1	0.014422
Effort	231.5680	1	< 2.2e-16
Reward	64.6605	1	8.898e-16
Preference:Effort	15.3293	1	9.031e-05
Preference:Reward	0.6845	1	0.408052
Effort:Reward	8.6731	1	0.003229
Preference:Effort:Reward	1.3302	1	0.248762

Note. With the choice coded as a binary outcome variable, preference, effort, reward and their interactions were defined as fixed effects whereby a random slope on the main effect of reward per participant was additionally included. All predictors were coded as continuous variables. A subject-level random intercept was included. AIC = 1238.5; BIC = 1304.6

Supplementary Table S7

Analysis of deviance (Type II Wald chi-square test) from the linear mixed-effects model on reaction time data for accepted work offers, with trial-by-trial preference as predictor, additionally including a random slope on reward

Predictor	χ^2	df	*p*-value
Preference	0.6212	1	0.4306045
Effort	29.6639	1	5.138e-08
Reward	14.0991	1	0.0001734
Preference:Effort	3.7048	1	0.0542569
Preference:Reward	0.0827	1	0.7736525
Effort:Reward	1.3089	1	0.2525867
Preference:Effort:Reward	0.3188	1	0.5723529

Note. With the reaction time coded as a continuous outcome variable, preference, effort, reward and their interactions were defined as fixed effects whereby a random slope on the main effect of reward per participant was additionally included. Preference was coded as a binary variable while effort and reward were coded as continuous variables. A subject-level random intercept was included. AIC = -336.9; BIC = -266.6

Supplementary Table S8

Analysis of deviance (Type II Wald chi-square test) from the linear mixed-effects model on reaction time data for accepted work offers, with general preference as predictor, additionally including a random slope on reward

Predictor	χ^2	df	*p*-value
Preference	0.3577	1	0.5497971
Effort	30.2882	1	3.724e-08
Reward	14.7564	1	0.0001223
Preference:Effort	8.3775	1	0.0037989
Preference:Reward	0.1087	1	0.7416191
Effort:Reward	1.5522	1	0.2128133
Preference:Effort:Reward	0.1206	1	0.7284348

Note. With the reaction time coded as a continuous outcome variable, preference, effort, reward and their interactions were defined as fixed effects whereby a random slope on the main effect of reward per participant was additionally included. All predictors were coded as continuous variables. A subject-level random intercept was included. AIC = -340.9; BIC = -270.5
